# AI Ecosystems for Human Flourishing: The Recommendations

**DOI:** 10.1007/978-3-030-69978-9_7

**Published:** 2021-03-18

**Authors:** Bernd Carsten Stahl

**Affiliations:** Centre for Computing and Social Responsibility, De Montfort University, Leicester, UK

**Keywords:** Ethical governance of AI, Requirements for ethical AI, Excellence and flourishing, Stakeholder engagement, Regulation and enforcement

## Abstract

This chapter develops the conclusions that can be drawn from the application of the ecosystem metaphor to AI. It highlights the challenges that arise for the ethical governance of AI ecosystems. These provide the basis for the definition of requirements that successful governance interventions have to fulfil. Three main requirements become apparent: the need for a clear delimitation of the boundaries of the ecosystem in question, the provision and maintenance of knowledge and capacities within the ecosystem, and the need for adaptable, flexible and careful governance structures that are capable of reacting to environmental changes. Based on these requirements, the chapter then spells out some recommendations for interventions that are likely to be able to shape AI ecosystems in ways that are conducive to human flourishing.

There are good reasons for thinking of AI in terms of ecosystems, as shown in Chapter 10.1007/978-3-030-69978-9_5. There are also good reasons for thinking of AI *ethics* in terms of ecosystems, as shown in Chapter 10.1007/978-3-030-69978-9_6. What remains is the task of translating insights into practical interventions that render the AI ecosystem conducive to human flourishing while taking into account the conceptual and empirical findings presented in Chapters 10.1007/978-3-030-69978-9_3 and 10.1007/978-3-030-69978-9_4. That is the task undertaken in this chapter.

## Challenges of Ethical Governance of the AI Ecosystem

Let us start with insights gained from the empirical research described earlier. As outlined in Chapter 10.1007/978-3-030-69978-9_2, there are competing interpretations of the concept of AI and varying views on why and how the technologies that are grouped under this label should be used. Any position taken on these concepts strongly influences the types of ethical issues that are associated with AI. For instance, machine learning has particular sets of characteristics that raise particular concerns, which are different from those raised by a wider understanding of AI as a socio-technical system with society-wide implications. Artificial general intelligence raises another set of concerns again. The multiplicity of concepts, issues, actions and actors is the motivation behind the choice of the ecosystem metaphor to describe the AI system.

What we can learn from this way of looking at AI is that any intervention at the level of the ecosystem must remain sensitive to this complexity. It must incorporate different understandings of the concepts involved, and take into account the role of and impact on the various stakeholders and the interplay between stakeholders, issues and interventions.

The problem is not only that there are many different issues, actors and responses. The ecosystem metaphor highlights the fact that the relationships between the constituent components of the system are often highly non-linear. This means that we can rarely expect to find simple cause-effect relationships. An intervention at some point of the ecosystem can have unexpected consequences that may have the opposite effect to that intended. This is a well-described phenomenon in natural ecosystems (Tenner 1997) that can be observed in similar ways in socio-technical systems, including AI ecosystems. These systems-related effects contribute to the general problem of unintended consequences.

The idea of intervening in an AI ecosystem in order to promote human flourishing is furthermore complicated by the often unclear and shifting boundaries of ecosystems. The boundary of an ecosystem is at least partly determined by the observer who is taking an interest in the system. Natural ecosystems can provide a good example. We could look at the entire earth as an ecosystem, but this can also be broken down into sub-systems, for example by geographical boundaries, which can again be broken down further, for instance by only looking at the habitat of one species. The value of a particular definition of a system with specified borders depends on what the observer who draws the boundaries wants to achieve.

Similarly, the AI ecosystem is not just one system but a system of systems of systems. For instance, we could look at the *global* AI ecosystem. There are some aspects of AI that are indeed global, notably the principles, techniques and technologies, and some of the dominant companies that have a global reach and presence. At the same time one can distinguish regional differences, e.g. between the USA, Europe and China, which could be described as separate ecosystems (see the discussion of different purposes of AI use in Section 3.3). The differentiation by geography and jurisdiction could go further, with, for example, the German system being different from the Spanish one, as shown by Kriechgaum et al. (2018) using the example of the innovation ecosystems surrounding photovoltaics. One can also differentiate further between AI ecosystems for different sectors or areas of application, such as autonomous transport, education, production and agriculture.

All AI ecosystems are embedded in environments which partly shape them but in turn are shaped by them. This throws up further challenges of governance, as any intervention tries to hit moving, interconnected targets. These environments cover technical, policy, economic, legal, social, ethical and other aspects that closely interact with AI and are very influential in the way ethical and related issues materialise, are perceived and can be addressed. They raise problems because their rate of change is likely to be different from that of the AI ecosystem.

As an example, let us look at the legal system, and more specifically at legal liability. Legal liability rules for AI are likely to have a significant impact on the way societies deal with AI. It is therefore not surprising that there are several reviews and recommendations at the European level alone which reflect on the applicability and possible development of liability legislation to render it suitable for AI (Expert Group on Liability and New Technologies 2019, European Commission 2020a, European Parliament 2020a). Liability legislation could therefore be considered a component of the AI ecosystem. At the same time, apart from existing black-letter law, there are also common-law and other legal practices and experiences. Legal professionals with expertise in liability do not necessarily have expertise in AI. Hence, there are different expectations from different fields of application of AI that will conceptualise liability differently. The interaction between the AI ecosystem (with its sub-systems) and the legal liability regime is likely to be complex.

Similar constellations are likely to be relevant to other social or technical systems. Let us take the technical system as an example: AI relies on existing and future ICT infrastructure, such as networking, computing and storage capacity. Progress in these areas has been a crucial factor in the success of machine learning. The availability of appropriate energy sources is a technical challenge but increasingly also a social, political and environmental one, due to the ever-increasing power consumption of AI systems and the potential interference with sustainability goals (Knight 2020). The AI ecosystem is thus crucially dependent on the technical infrastructure, and drives and shapes its development. But decisions about the technical infrastructure are not necessarily taken by members of the AI ecosystem and can therefore appear to be part of the external environment. Evaluation of the state of the ecosystem and perceptions of its progress, potential and ability to change will therefore depend heavily on where exactly the boundary is drawn.

A further key challenge for the ethical governance of AI ecosystems is the concept of ethics. In Chapter 10.1007/978-3-030-69978-9_2 I proposed the concept of human flourishing as the concept to guide the understanding of ethics in AI. “Flourishing” is a well-established term strongly linked to the ancient tradition of virtue ethics; it is an inclusive term that is open to figures of thought from other philosophical traditions, such as utility, duty and care. At the same time this openness can be problematic because it is difficult to determine when and how exactly flourishing has been achieved (see box).

### Determining Flourishing

One approach that aims for human flourishing, and simultaneously tries to provide concrete guidance on how to measure conditions for flourishing, was developed by Martha Nussbaum: the capabilities approach (Nussbaum 2000, Buckingham n.d.). The ten capabilities required for flourishing, according to Nussbaum, are life; bodily health; bodily integrity; senses, imagination and thought; emotions; practical reason; affiliation; other species (e.g. connection to animals and nature); play; and control over one’s environment (Nussbaum 2000: 78–80). Economists have taken Nussbaum’s work and assessed whether capabilities and related interventions can be reliably measured (Anand et al 2008). Their conclusion is that economic models focused on Nussbaum’s capabilities *can* measure and address some inhibitors of human flourishing, but not all (ibid. 303) due to the fact that capabilities have multiple dimensions (ibid. 302).

We therefore cannot assume that there is an a priori way of determining whether people are flourishing, so we need to concede that this is at least partly an empirical matter which is also subject to change over time. People’s moral perceptions and positions change, while AI ecosystems are realised within the shifting boundaries of ethical preferences. At present this may best be illustrated using the different privacy and data protection regimes in different parts of the world, which arguably reflect different social preferences and give rise to interesting debates about what, if anything, is universal and should be applied across geographical and other boundaries. For instance, the right to privacy is recognised as a human right in the European Convention on Human Rights, which provides a strong basis for data protection as a crucial component of safeguarding informational privacy. In the EU data protection is regulated through the General Data Protection Regulation, which provides detailed guidance and requires certain activities and procedures, such as the need to have a legal basis for the processing of data and requirements to undertake data protection impact assessments and appoint data protection officers. The European emphasis on data protection is likely to strongly influence how AI will be regulated (EDPS 2020). In other parts of the world privacy and data protection have different roles and relevance. While data protection legislation exists in many jurisdictions, its extent and enforcement varies. In China, for example, privacy laws protect citizens’ data from abuse by third parties, but they do not cover governmental data access and use (Gal 2020).

The concept of human flourishing has some universal claims, notably that humans strive for happiness and that achieving this is an ethically justified aim that societies and the technologies they employ ought to support. But how exactly this is achieved and how we can know whether it has been achieved remain open questions. And this openness is not just a historically contingent fact, but part of the question itself. It is not a question that one can expect to answer and close, but one that needs ongoing reflection and discussion, as particular answers differ and vary over time.

This also implies another major challenge to the ethical governance of AI ecosystems, namely the inevitable existence of ethical disagreement and value conflicts. As part of the process of reflecting on and promoting human flourishing, people will come into conflict. Conflicts may be local, for example where scarce resources must be allocated to satisfy competing demands. A typical example would be the use of water to keep golf courses green versus other uses (Scott et al. 2018). But they can also be more fundamental, where ethically well-justified positions come into conflict and resolutions of such conflicts are not obvious and straightforward. An example might be the controversy over mobile tracking and tracing apps during the COVID-19 crisis, in which competing demands from privacy campaigners and public health experts have led to a number of controversies over how such technologies could and should be used to limit the spread of the disease (Klar and Lanzerath 2020). This is also a good example of the problems in drawing a boundary around a socio-technical innovation ecosystem in terms of jurisdictions, data, technical platform etc.

The final challenge to the successful ethical governance of AI ecosystems is the uncertainty of all aspects of the ecosystems themselves and of their environments, be they technical, social or ethical. Technical uncertainty may be the most visible example, with AI-related technical developments happening at a rapid rate, which renders the value of trying to predict the next step exceedingly limited. This is partly a function of the technology itself, but partly also a function of the growing realisation of the potential applications of such technologies. The application of current machine learning technologies may lead to radical changes in coming years even without any further technical progress, simply because actors are beginning to understand what these technologies can do and to apply them to new problems in novel ways.

But the uncertainty of the future is not just linked to technical artefacts. It is equally important in terms of social structures and ethical preferences. Societies are always dynamic, and this can play out in ways that affect technological ecosystems in unpredictable ways. Again, the COVID-19 pandemic can serve as an illustration of the sometimes rapid change of social systems. Widespread working from home may be supported by AI technologies to the benefit of employees, but it can also offer new modes of surveillance and exploitation of workers (Harwell 2020). As another example, the heightened awareness of racism that has arisen in the context of the Black Lives Matter movement has put an even stronger spotlight on bias and discrimination, already a well-discussed topic of AI ethics. While the potential of AI to lead to bias and discrimination is well established, it has also been remarked that it may turn out to be a useful tool in identifying existing human biases and thereby overcoming them (Stone et al. 2016). It is impossible to predict which social change will raise the next set of challenges and how the interaction between the AI ecosystem and other parts of our social and technical environment will develop.

**Fig. 7.1:**
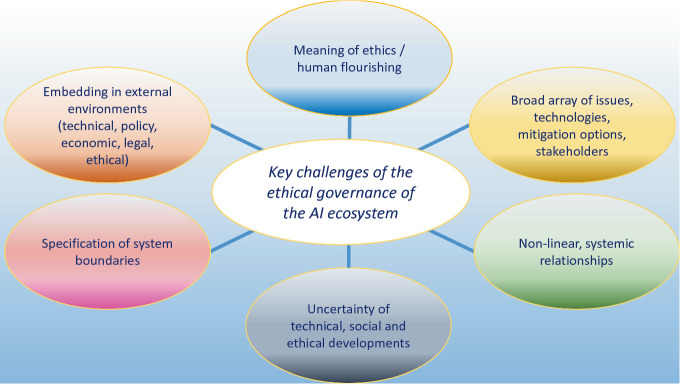
Key challenges of ethical governance of AI ecosystems

Figure 7.1 summarises the points discussed in this section, in which I have indicated that most of the key challenges are not specific to AI. Some of them arise from the systemic nature of the socio-technical innovation ecosystem. Some of them are related to fundamental aspects of the social, technical and natural world we live in. AI-specific issues that are linked to the characteristics of the underlying technologies and their impact on the world form only a sub-set of these challenges. This indicates that the governance of AI ecosystems is best understood as a part of the governance of digital technologies, which, in turn, is a sub-set of technology governance overall.

Having now explored the challenges that any attempt to govern AI ecosystems to support human flourishing is likely to face, we can move on to the next step, an exploration of what any intervention would need to cover, in order to address the challenges discussed here.

## Requirements for Shaping the AI Ecosystem

Interventions designed to address the ethical challenges of AI can be developed in a multitude of ways. The way chosen here is to situate interventions within the AI ecosystem metaphor. A number of requirements for this to work are outlined below.

### Clear Boundary of the Ecosystem

The first requirement highlights the importance of drawing clear boundaries around the ecosystem that is to be targeted. This refers to the geographical, technical, cultural and other boundaries that determine what is included in the ecosystem. Boundaries of an ecosystem, as indicated earlier, are not so much natural phenomena as the result of human decisions.

In some cases, these boundaries may seem obvious to the actors involved. In the case of the European Union’s discussion of AI, one implicit assumption is that any intervention that the EU undertakes will be at the European level and fall within the EU’s jurisdiction. This of course makes perfect sense for legal institutions that work in a defined jurisdiction and therefore intervene within the boundaries of that jurisdiction. The same is true for national regulatory interventions, which are normally aimed at the members of the ecosystem that are active within a nation’s borders.

However, it is also clear that the application of jurisdictional boundaries to AI ecosystems is not necessarily the most promising approach. AI principles, the underlying science and algorithms are not locally confined. Many of the key companies in the field are global. The potential mismatch between regional regulation and global technology is not new and not confined to AI (Xu et al. 2004). It is worth being aware of and explicit about it, however, to ensure that the expectations levelled at interventions are realistic.

A similar challenge to the setting of clear boundaries around relevant ecosystems is the terminology describing the underlying technology. As I showed in Chapter 10.1007/978-3-030-69978-9_2, the challenge of defining AI means that it is difficult to determine which ethical issues, or which possible mitigations, are relevant. When describing the AI ecosystem, do we refer to AI in a narrow sense, notably to questions of machine learning, neural network, deep learning etc.? Or do we include other aspects of AI, such as fuzzy logic and expert systems? Or do we include the broader socio-technical systems that may or may not embrace narrow AI somewhere along the value chain?

Focusing on AI in a narrow sense has the advantage that technologies are more easily described. The characteristics that give rise to concerns can be identified and a set of ethical issues can be determined. In many cases, the narrower focus might make it easier to find a resolution. A good example of this would be biases that machine learning algorithms pick up from existing datasets which lead to unfair discrimination. This is by now a well-recognised problem and much work is being undertaken to find ways of addressing it (Holzinger et al. 2017).

While such a narrow view of the technologies that constitute the AI ecosystem is thus suitable for resolving particular issues, it is arguably not helpful if one seeks to arrive at a more comprehensive approach that covers the breadth of the current AI ethics discourse. Most of the issues that arise from living in a digitally enabled society go beyond specific technologies and easily identifiable causal chains. While many concerns about fairness, the distribution of opportunities and burdens, employment etc. are related to narrow AI, they typically go beyond the immediate technology used.

The struggle with the question of how to draw the ecosystem boundary can be observed at the European level. For instance, the European Commision’s White Paper on Artificial Intelligence (2020c) speaks only of AI and seems to focus on machine learning, but it also refers to other technologies such as quantum computing. This means that no boundary was determined: AI was not defined or set apart from other technologies. In a different report on the safety and liability implications of AI (European Commission 2020a), the technical scope was already broadened in the title to include “the internet of things and robotics”. This means that there is a lack of agreement on the exact scope and delimitations of the term even within a single policymaking body such as the European Commission. In its policy outline for Europe’s digital future (European Commission 2020b), the authors use the concept of “deep tech”, which includes supercomputing, quantum technologies, blockchain and secure pan-European cloud capacities.

This shows the difficulty of clearly delimiting which technologies are of relevance in any given debate or for given reports. One could instead use the term “ smart information systems” (SIS), defining SIS as those socio-technical systems that have as part of their core capacities narrow AI and big data analytics but also include other technologies to collect and process data and interact with the external environment. (Stahl and Wright 2018). This is a useful term, but in the public and policy discourse the term “AI” has become dominant. For the purposes of the delimitation of the ecosystem it is nevertheless important to make clear which technologies are covered and which, by implication, are not.

A third aspect of drawing clear boundaries for the ecosystem, in addition to geographical boundaries and technical terminology, involves its normative aspects. These start with the normative assumptions behind the definition of the ecosystem. Decisions on the geographical, technical and social boundaries of an ecosystem are based on underlying assumptions and values that must be made explicit. What is the observer of the ecosystem who uses the ecosystem metaphor trying to achieve? In Chapter 10.1007/978-3-030-69978-9_4 I suggested that there are different purposes that drive the development and use of AI (economic efficiency, social control, human flourishing) and that the delimitation of the ecosystem should include a clarification of which of these (or maybe other) purposes motivate the description of the ecosystem.

If the purpose of using the ecosystem metaphor is to find ways of promoting human flourishing, then this should not only be explicit, but also come with a health warning. Ethical questions are not subject to straightforward resolutions. Anyone explicitly attempting to promote ethics would be well advised to proactively engage in expectation management. Promising to solve the ethics of AI is unlikely to be successful on all counts and may therefore result in disappointment and disillusionment. It might therefore be more fruitful to focus on specific indicators of how human flourishing can be promoted. This may be achieved, for example, by focusing on how some or all the fundamental human rights could be strengthened in an AI ecosystem or by referring to how the AI ecosystem would promote the achievement of the UN’s Sustainable Development Goals (SDGs). As such the boundary setting implied in declaring that human flourishing is the ethics focus of the AI ecosystem is narrowed down to specific goals related to promoting human rights.

Defining and delimiting the AI ecosystem in terms of geographical, jurisdictional, cultural or other boundaries, and clarifying the technologies to be covered and the normative aims that are to be achieved, constitute an important first step for a successful intervention in such an AI ecosystem. But on its own this step cannot make a difference.

The next question, then, is: what is required to shape this ecosystem to support human flourishing?

### Knowledge and Capacity

One of the characteristic features of innovation ecosystems is that the members of the system not only compete *and* cooperate, but also co-evolve and learn from one another. The existence and availability of knowledge are key factors distinguishing different ecosystems. Knowledge also plays a key role in understanding ethical questions and ways of addressing them. The range and quality of knowledge within an ecosystem are therefore key factors affecting the ability to understand and address ethical concerns.

Shaping an AI ecosystem in a way that promotes human flourishing requires and builds on knowledge. This claim is uncontentious for the technical and economic side of AI ecosystems. The hotbeds of current AI development, notably key geographical areas such as Silicon Valley, are characterised by high levels of available technical talent and knowledge concentrated in high-profile universities and companies. Similarly, building up this technical knowledge base is a key component of most national AI strategies related to particular economies. Such knowledge includes technical knowledge in the narrow sense, but also the knowledge of business processes, finance options and so on that is required for AI organisations to operate successfully.

The same is true for the wider non-technical or normative knowledge that shapes the AI ecosystem. This knowledge covers much of what I have discussed in earlier chapters, such as various concepts of ethics, the ethical issues that are typically associated with AI and the various mitigation options that have been proposed and are being discussed. An ecosystem can only be shaped to promote human flourishing when significant knowledge of ethical issues and potential solutions to ethical challenges is available.

In addition, there is a need for the procedural knowledge that is required to address ethical issues. This is knowledge of how to organise the processes that are required to deal with ethical questions. For instance, if discrimination possibilities are to be reduced to increase human flourishing, the governance options to achieve this need to be known, from legal solutions to technical solutions.

Procedural knowledge should also cover other aspects of reflecting on and evaluating science and technology. Drawing on the discourse on responsible research and innovation, one can identify some important processes that may contribute to ensuring that ethical issues can be recognised and dealt with. These include anticipation, engagement, reflexivity and responsiveness (Stilgoe et al. 2013).

Processes of anticipation are required for a structured way of thinking about possible future states that will inform the way in which we act today and prepare for the future. These processes should not be misinterpreted as simple predictions that try to guess what the future will look like. Accurate predictions are notoriously difficult, if not impossible, and the literature is littered with predictions that have turned out to be wrong and in hindsight tend to look ridiculous, such as the prediction by the president of IBM in 1943 that there would be a world market for maybe five computers, or the statement by the chairman of Digital Equipment Corporation in 1977 that there was no reason for anyone to want a computer in their home (Himanen 2001: 187). Processes of anticipation are based on the recognition of the impossibility of prediction. They nevertheless aim to explore possible futures, to help societies decide which actions to take today (Cuhls 2003). There are well-established discourses and academic disciplines that have developed methods for future and foresight studies (Sardar 2010), some of which explicitly focus on the ethical issues of emerging technologies (Brey 2011, Floridi and Strait 2020). For instance, Flick et al. (2020) explore a wide range of resources, including academic publications, but also social media discussions, to identify expected technical developments in the field of ICT for ageing and ethical concerns that may arise from these. This type of work opens up spaces of possibilities without committing itself to one particular outcome. It is useful in raising awareness and sensitivity to both technical and social or ethical developments and therefore offers the likelihood that these can be shaped appropriately. This type of work can benefit AI ecosystems, but for this to happen, the knowledge of how to undertake anticipatory work needs to be available within the ecosystem.

One of the processes with the potential to draw knowledge into the AI ethics ecosystem is the engagement of all stakeholders. “Engagement” refers to activities that bring together different stakeholders in an open manner for a mutually informative exchange of ideas. The importance of engagement in science, research and technology development is long established (Arnstein 1969, Hart et al. 2009, Bickerstaff et al. 2010, Van Est 2011, Boulton et al. 2012). Certain aspects of engagement are also well established in technical disciplines, for example in the form of user engagement or user experience research, which form part of computer science, the parent discipline of AI (Haenlein and Kaplan 2019). However, in order to undertake engagement activities in a way that is ethically sensitive and can contribute to an AI ecosystem so as to promote human flourishing, they need to be employed carefully. Engagement in science and technology development is sometimes limited to exercises for the public understanding of science, which aim to inform the public about scientific insights or technical achievements. There is nothing wrong with such exercises, but they are only one part of public engagement, which, in order to live up to ethical expectations, needs to facilitate and open two-way communication, with researchers and other stakeholders being willing to engage, listen and respond to each other and take positions seriously. If this is not done in an inclusive manner, important knowledge to be gained about the AI ethics ecosystem might be lost.

While such an open engagement process promises both better understanding of the ecosystem through a broadening of the knowledge base and higher levels of acceptability of the resulting research and technologies, there is no guarantee that these will be achieved. Public debates about science, research and technology in many other areas, such as genetically modified organisms, nuclear energy and nanotechnology, show that engagement activities can be highly charged and conflictual (Van Est 2011). The potential for fundamental disagreements on underlying values, aims or desired outcomes pervades all such stakeholder engagements, whether high-profile at a national or international level or conducted at a local or organisational level.

All of these different aspects of knowledge need to exist in a practical and applicable form. It is not sufficient to have them in repositories that are not accessible or not used. Among the requirements for shaping AI ecosystems is thus that the knowledge base be accompanied by and to a large extent realised by a corresponding capacity to *apply* the knowledge. Capacity building is therefore a further key requirement: the different stakeholders need to not only recognise the legitimacy of different types of knowledge but be willing to engage with different knowledges and, ideally, develop their own capacities in applying these different knowledges. As Coeckelbergh (2020: 179) puts it, “if engineers learn to do things with texts and humanities people learn to do things with computers, there is more hope for a technology ethics and policy that works in practice”. Of course, there are other stakeholders involved in AI ecosystems besides engineers and humanities specialists, and other knowledge domains besides “things with text” and “things with computers”. But the general sentiment, that people need to be willing to gain new insights and learn new skills, is undoubtedly true.

The question of how this may be achieved brings us to the third group of requirements for shaping an AI ecosystem for human flourishing: the question of system governance.

### Governance Principles of AI Ecosystems

The characteristics of innovation ecosystems and the resulting challenges for shaping AI ecosystems to promote human flourishing call for approaches to the governance of these systems that are sensitive to them. I am using the term “governance” here to denote all activities and processes that are put in place to facilitate and regulate the behaviour of the members of the AI ecosystem and the relationship of the ecosystem to its broader environment. The term frequently refers to structures and processes within organisations, whereas at a higher level the term “regulation” is used (Braithwaite and Drahos 2000). However, “governance” is increasingly used to describe a much broader array of

processes of governing, whether undertaken by a government, market, or network, whether over a family, tribe, formal or informal organization, or territory, and whether through laws, norms, power, or language. (Bevir 2012: 1)

The term also refers to specific localised ways of organising (or governing) particular issues, as in data governance (Khatri and Brown 2010) or information governance (ISO 2008), rendering them suitable to describe ways of dealing with AI ecosystems that cover many societal actors and activities.

A key requirement for the governance of AI ecosystems is flexibility. We have seen that the members of an AI ecosystem are in complex and non-linear relationships. In addition, the technologies, organisations, social dynamics and other aspects of the ecosystem can change rapidly. Any governance structure therefore needs to be able to react flexibly to change. Kuhlmann et al. (2019) use the term “tentative governance” to describe this flexibility. They consider governance to be tentative

when it is designed, practiced, exercised or evolves as a dynamic process to manage interdependencies and contingencies in a *non*-*finalizing* way; it is prudent (e.g. involving trial and error, or learning processes in general) and preliminary (e.g. temporally limited) rather than assertive and persistent. Tentative governance actors seek flexibility and act incrementally. (Kuhlmann et al. 2019: 1093, emphasis in original).

Such tentative governance needs to provide spaces for actors to learn and develop understanding of technologies, their use and their evaluation. It must be based on and foster communication between stakeholders. It should also allow for the acknowledgement of mistakes and have the ability to reverse or change course where initial assumptions prove to be wrong or where new insights or consensus emerge.

AI ecosystem governance should also be open to conflict and disagreement and be able to deal with those constructively. As Genus and Stirling (2018) rightly point out, responsible engagement with technology requires what they call “Collingridge qualities” (see Collingridge 1981), namely inclusion, openness, diversity, incrementalism, flexibility and reversibility. In many cases these can be better helped by exploring disagreement and dissensus than by engineering consensus.

The governance of AI ecosystems should be sensitive to the motivations and incentives of the members of the ecosystem. It needs to carefully balance the possible and expected benefits of AI with the possible and expected downsides. This requires an ability to draw on the knowledge and capacity described earlier, to evaluate developments and to put in place incentives and sanctions that reinforce developments that are desirable and promote human flourishing.

The AI ecosystem does not exist in a vacuum, and its governance should therefore be linked to existing governance structures. Questions regarding the extension of liability legislation to allow it to cover AI as currently discussed at the European level and elsewhere are one category of questions related to the extension of existing governance structures to include AI.

The consideration of existing governance structures is important to ensure the consistency of overlapping governance regimes, which, in turn, is important for the success of any governance efforts. Elsewhere (Stahl 2013, Stahl et al. 2019) I have introduced the concept of meta-responsibility as an important part of responsible research and innovation (RRI). This idea arises from the existence of networks of responsibility (Timmermans et al. 2017) which govern the practices of science, research and innovation. RRI as a meta-responsibility aims to shape, maintain, develop, coordinate and align existing and novel research- and innovation-related processes, actors and responsibilities with a view to ensuring desirable and acceptable research outcomes. This idea is relevant to AI ecosystems as well. AI ecosystems build on and incorporate many existing responsibilities and governance structures. In order for these ecosystems to be successful and to promote human flourishing, it is not necessary to re-invent principles of governance; they should rather be carefully developed, to help existing governance structures and responsibility relationships work effectively. In order to promote AI ecosystems that are conducive to human flourishing, we do not need to re-invent the wheel, but we need to make sure that the many wheels that already exist point in a roughly similar direction and that there is a strong and legitimate process that allows this direction to be determined.

**Fig. 7.2:**
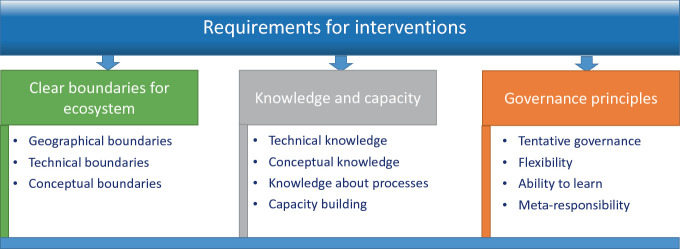
Requirements for interventions into AI ecosystems

Figure 7.2 presents a summary of the main points discussed in this section and aims to answer the question: which characteristics should an intervention into AI ecosystems have, to be likely to deal with ethical aspects successfully? These are necessary requirements, but may well not be the only ones, and are very unlikely to be sufficient on their own. They should nevertheless be useful in reviewing and evaluating practical interventions, policies and governance structures and could help to improve those. They thus contribute to the types of recommendations which I outline in the next section.

## Shaping AI Ecosystems

The proposals that follow are not ready-made recommendations that can be implemented as is to solve the ethical issues of AI. Apart from the fact that this is impossible because ethical issues do not normally lend themselves to simple resolution, I also lack the space in this book and the detailed knowledge of the various domains of expertise that would be required to develop detailed implementation plans.

What I am trying to do is to highlight some key aspects of governance, mitigation and interventions that have a high likelihood of making a positive contribution to the aim of shaping AI ecosystems in desired ways.

The audience envisaged for these proposals includes decision-makers who can shape aspects of the AI ecosystem, and also people who have an interest in and form part of the debate: policymakers as well as researchers and users of AI in public and private organisations, the media and civil society representatives.

AI ecosystems exist world-wide but are often geographically defined, subject to the laws of a particular jurisdiction, and can be sector-specific. My suggestions aim to be broadly applicable across geographies and jurisdictions, but they should be checked for local applicability. Moreover, most of the work that underpins my thinking was done in the UK and EU contexts, funded by European research grants. Many of the questions that influence how AI ecosystems can and will be shaped are being hotly debated at the time of writing (the European summer of 2020). At the European level in particular there is a set of proposals from the European Commission, the European Parliament and high-level expert groups. While this discussion is therefore clearly tilted towards Europe, I believe that the principles outlined are valid – or at least of interest – beyond Europe.

The sub-sections below focus on proposals for actions intended to ensure that AI ecosystems are conducive to human flourishing.

### Conceptual Clarification: Move Beyond AI

Any successful intervention that promotes human flourishing in AI ecosystems needs to clarify which concept of AI is being used. While this is trivially obvious, it is also a difficult suggestion to implement. The concept of AI remains difficult to define and, as my discussion in Chapter 10.1007/978-3-030-69978-9_2 indicates, that there are fundamentally different technical artefacts and socio-technical systems. I am not suggesting that it would be possible to achieve conceptual divergence by decree. Rather, I believe that any intervention that aims to affect AI ecosystems must state clearly what it intends to cover. The multitude of meanings of AI and the lack of an agreed definition throw up serious doubts about the possibility of regulating AI (Stone et al. 2016).

Depending on the chosen concept of AI, it is possible that only a very specific part of the ecosystem will be affected, and the consequences for the overall ecosystem may be limited. For example, interventions that aim at a particular set of technologies, such as machine learning, will be limited to addressing effects that are clearly linked to these technologies, such as algorithmic biases or biases that arise from the development of models based on data containing biases. Choosing a narrow area of AI has the advantage of allowing for the definition of a closely circumscribed target for an intervention which then has a high likelihood of being successfully implemented. The disadvantage of such an approach is that interventions of this kind are not likely to have a major effect across broader AI ecosystems that are based on broader definitions of AI. The current approach by the European Commission (2020c) as outlined in its White Paper seems to pursue this strategy. While the definitions of AI in the document are broader, the aim seems to be to address the specifics of machine learning. My claim here is that the definition and the target of the intervention need to align.

The clear use of terminology is thus an important recommendation for anybody aiming to intervene in an AI ecosystem. However, competing understandings of the term “ artificial intelligence” might lead to confusion. This is partly owing to the varying definitions, but also partly to the emotive connotations of AI. Humans tend to think of themselves as intelligent, and AI therefore has the potential to threaten our perceptions of ourselves. Popular culture and fiction have developed a range of easily recognisable tropes of AI, such as the evil robot, which colour the perception of actual and expected technologies. There can be little doubt that this is one of the causes of the high profile of the AI debate, but it is also its Achilles heel.

As a general suggestion I would therefore advocate using the term AI sparingly and moving beyond it to terms that better capture the particular technology or technologies in question. If an intervention is aimed at a specific aspect of AI, such as machine learning, then that should be made explicit. If the aim is to cover the broader ecosystem and address issues arising from life in a digital world, then a broader term would be more suitable.

My suggestion would be to speak of something like “emerging digital technologies”, which probably covers the broad range of technologies of potential relevance, from narrow AI to neuromorphic computing, quantum computing, the internet of things, robotics and future digital technologies. This term will of course still have to be clearly defined, but the word “emerging” indicates that it is a moving target. It is also a much less emotive term than “ artificial intelligence”, probably not as threatening and likely to attract less attention. Considering the current hype around AI, I would suggest that a lower level of attention might be a good thing, as it might allow more careful deliberation in the planning of possible interventions.

### Excellence and Flourishing: Recognise Their Interdependence

The previous suggestion pointed to the delimitation of the AI ecosystem in terms of the concepts and the technologies involved, whereas this one focuses on the conceptual clarity of the normative dimension. An intervention in an AI ecosystem requires the clarification of the purpose of the intervention: what is the intended outcome and why is the intervention deemed desirable?

In Chapter 10.1007/978-3-030-69978-9_3 I discussed the different purposes of AI that pervade much of the AI policy literature: economic benefits, social control and human flourishing. While this is an artificial and analytic distinction, I believe that a clear statement of purpose would be helpful for most interventions.

An intervention into an AI ecosystem for ethical purposes should explicitly state the ethical intention. I have advanced the concept of human flourishing as a broad term covering many different ethical positions and traditions. However, there are many other terms that could denote similar aims, such as fairness, equality, dignity and more specific aims such as the pursuit of the SDGs or the promotion of human rights. The point is that such a commitment is important to ensure that the intervention into the ecosystem can be monitored and assessed accordingly, and it should therefore be made explicit.

An important aspect of the commitment to an ethical position is its relationship to the technical environment. There is broad agreement that national and regional policymakers have a role in developing the technical AI ecosystem. That role tends to include measures such as the strengthening of educational pathways leading to an AI-proficient workforce, support for venture capital and other means of financing new AI-driven business models, and research and innovation structures that provide funding for AI-related research. The strengthening of the AI ecosystem is often also seen as requiring the creation of national or regional AI champions or centres of excellence that serve as hubs to bring together knowledge and expertise. In the UK this role is fulfilled by the Alan Turing Institute. The European Commission proposes the creation of networks of excellence at leading universities for a similar purpose.

From my perspective it is important to establish that the ethical dimension should be understood as an integral part of the technical AI ecosystem, not as an add-on. For AI to become truly geared towards human flourishing, it must overcome the current division between scientific excellence and ethical reflection and recognise that scientific excellence cannot be truly excellent if it does not consider the social and ethical consequences of the development, deployment and use of the technology.

At first sight, this might sound rather trivial, but it would require a far-reaching reconceptualisation of ethics in science, research and technology development. At present, ethics in research and innovation tends to be focused on research ethics, which typically takes the form of a review at the start of a project on the basis of biomedical research ethics principles. My suggestion is to fundamentally rethink the relationship of AI research and innovation and ethics. Ethics in the broad sense of promoting human flourishing should be an integral part of scientific excellence. This would mean that aspects such as intended consequences, detailed risk analysis and contingency plans that cover known or expected ethical issues would form part of the scientific evaluation of proposals and determine which ideas are seen as excellent and thus worthy of being funded.

### Measurements of Flourishing: Understanding Expected Impacts

An important ingredient for ensuring that AI ecosystems drive towards human flourishing is the ability to reflect on the successes of prior interventions and use them as a basis for steering the ecosystem. This requires an understanding of the intended and real impacts of the activities in the ecosystem. With regard to the desire to promote human flourishing, it calls for ways of understanding human flourishing in practice.

At present there is no agreed-upon methodology or set of criteria that can be used to assess how the consequences of the development and use of AI have affected or are affecting human flourishing. Or, to put it differently, we need to have measures for human flourishing and these need to be applicable to AI ecosystems. However, we do not need to start from scratch in developing such measurements.

There are well-accepted mechanisms that provide at least parts of what is required. One established method for measuring human flourishing is the Human Development Index (UNDP n.d.). This set of measures was inspired by the Capability Approach (Sen 1993, Alkire 2002, Nussbaum 2011), which set out to move beyond the measurement of gross domestic product and instead evaluate the quality of human development. It has been adopted by the United Nations Development Programme as a key measure and has also been successfully applied to ICT (Johnstone 2007, Zheng and Stahl 2011). Hence, it seems plausible that it could easily be tailored to cover the specific aspects of AI.

A similar set of ideas has driven the development of the SDGs, which currently seem more prominent with regard to AI, notably in the “ AI for Good” discourse (Taddeo and Floridi 2018). Orienting AI development, deployment and use towards the achievement of the SDGs is a key component of AI for Good as promoted by the International Telecommunications Union (ITU n.d.). The idea of moving towards the SDGs is currently not very contentious, even though in practice it may not be trivial and could lead to trade-offs between different ethically relevant goals (Ryan et al. 2020)

A further approach that also has the advantage of being based on recognised international agreements is the focus on human rights. As discussed in Chapter 10.1007/978-3-030-69978-9_4, there are already a number of proposals on finding ways of applying SDGs or human rights to AI (Raso et al. 2018, Latonero 2018, Commissioner for Human Rights 2019).

All of these approaches appear to have a high potential for being applicable to AI and providing ways to structure discussion and understanding of the impact of interventions in the AI ecosystem. A clearer understanding of their respective strengths and weaknesses would be helpful in deciding which ones might be most appropriate in which AI ecosystems.

The question of human flourishing and the influence that AI can have on this is not easy to resolve. The pointers in this section to the Human Development Index, the SDGs and human rights measures are meant to provide indications of how such influence may be achieved. Trying to measure something as complex and multi-faceted as flourishing raises many challenges. Some of these are methodological and epistemological, revolving around the questions: what can we measure and how can it be measured? The very term “measure” suggests a quantitative approach, and the degree to a complex qualitative term such as flourishing can be captured using quantitative measures is open to debate. The challenges go even further and touch on the heart of ethics, on the question: is it suitable at all to even try to measure human flourishing?

This book cannot offer a comprehensive answer to that question. However, it can point to the fact that we live in a world where measurements drive many policies and behaviours. An ability to express whether a particular aim, such as human flourishing, has been achieved, or whether an activity or process can lead to progress in the direction of this aim, would therefore help engage decision-makers who are used to this type of discourse. Developing measurements is highly ambitious, and it is very unlikely that we will ever be able to measure human flourishing comprehensively. But the benefits of having some, albeit imperfect, measures may well be worth the disagreements that these measures are likely to evoke.

### AI Benefits, Risks and Capabilities: Communication, Knowledge and Capacity Building

At the heart of any attempt to shape AI ecosystems and move them in the direction of human flourishing must be an understanding of the benefits and risks of the technologies and the capabilities they can bestow on users. The fast-moving nature of AI means that this knowledge may lose its currency quickly, which is why I suggest that an AI knowledge base is a requirement for the successful shaping of AI ecosystems.

Such a knowledge base would no doubt be based on existing structures of knowledge and learning, including academic publications and databases, and web resources. A key role in establishing and maintaining this knowledge base would be filled by the centres of excellence – those that are already established and the new centres or network structures that are being developed. In addition, several international organisations, such as the OECD, UNESCO and the European Commission, are developing databases, observatories etc. to capture the discourse. Standardisation bodies have an important role to play in collecting available knowledge and facilitating consensus on key aspects.

One key suggestion I would like to make in this respect mirrors the one in the section on excellence and flourishing (Section 7.3.2), namely, to ensure that no artificial divide is imposed between scientific knowledge and ethical and social understanding. This means that AI centres of excellence should include excellence in the ethics of AI, a position that the Alan Turing Institute in the UK, for example, has already adopted. Similarly, while there is no doubt scope for specialised standardisation on ethics and AI, as the IEEE P7000 family of standards shows, it would be desirable for technical AI standards to refer to and include ethical aspects.

The AI knowledge base needs to be scientifically sound and reliable, but it must also be visible, communicated and understood, which implies the need for educational activities, from primary education all the way up to post-doctoral work. This, in turn, calls for reviews of national and disciplinary curricula, the development of learning support and the creation of teaching capacity.

The further dissemination and practical societal usefulness of this knowledge will depend on whether it can be conveyed in a simple and comprehensible manner. One approach to this is to develop labels and certificates for AI systems, comparable to well-established labels such as those codifying energy consumption, the nutritional content of food and environmental sustainability. It may help to use existing categorisations of AI, such as the six levels of autonomy – levels 0 to 5 (SAE 2018) – that are used for autonomous vehicles to convey relevant aspects of benefit and risk. Such relatively simple categorisations of important aspects of AI may help visually represent possible benefits and risks and thus support balanced decision making.

### Stakeholder Engagement: Understanding Societal Preferences

The suggestions above assume that there is a position on AI that allows us to determine which uses and applications of technology are desirable and acceptable, that there is some sort of agreement on what counts as flourishing or which benefits warrant taking particular risks. While I believe that one can indeed find much consensus on many of these questions, at least within specific communities and states, there will always be new or borderline phenomena that are less clearly understood and give rise to different interpretations and evaluations.

The complexity of AI and other emerging digital technologies, both in terms of their technical capacities and in relation to societal outcomes and impact, means that it is unlikely that these questions will be easy to settle. Furthermore, in many cases they will fall into line with existing societal disagreements, e.g. with regard to what counts as just distribution or what a state can reasonably require its citizens to do.

A full understanding of what counts as an ethical issue related to AI, why it counts and what, if anything, could or should be done about it therefore calls for societal debates that allow stakeholders to come together and debate these questions. As a consequence, the ethics of AI cannot be a topic that is dealt with by technical and ethical experts alone: it calls for broader stakeholder engagement.

To a large extent the political processes that exist in a democratic state can take care of this task and provide means for the expression of divergent opinions and legitimate decisions concerning desirable actions. In order for AI ecosystems to be steered towards human flourishing, they will therefore need mechanisms that institutionalise stakeholder engagement activities that give stakeholders a voice and allow them to contribute meaningfully to collective decision-making. Appropriate recommendations and policies seem to call for a multi-stakeholder approach that brings together relevant stakeholders in an inclusive manner to move towards human flourishing or, as Cath et al. (2016: 18) put it, to deliver a “good AI society”.

This is much easier said than done. There are many potential pitfalls in stakeholder engagement. Such activities need to be carefully defined, planned and executed to avoid their being hijacked by particular interests (Wehling 2012). They need to be aligned with existing democratic processes. There are difficult questions about the frequency and intensity of stakeholder engagements which have to do with the costs they incur and whether they can truly claim to represent stakeholder opinions (Wynne 2006, Felt and Fochler 2010). Notwithstanding these potential problems and downsides, however, it is difficult to see how AI ecosystems can properly understand the ethical issues they face and acceptable ways of dealing with them unless they have appropriate ways of consulting stakeholders.

### Responsibility for Regulation and Enforcement: Defining the Central Node(s) of the AI Ecosystems

My last suggestion for shaping the AI ecosystem has a bearing on all the other suggestions. It relates to to the question of where responsibility lies for planning, realising, implementing and enforcing the suggestions made here and elsewhere. This refers to the concept of meta-responsibility, i.e. the question of who or what is responsible for ensuring that individuals, organisations and states understand their responsibilities and fulfil them.

I believe that a key condition for any suggestion or recommendation to be successful is the ability to answer the question: who or what is responsible for implementing it? At the national or international level this relates to the question of whether there should be a regulator for AI and what form it should take. At the European level we observe several opinions on this. The European Commission (2020a) is in favour of a strengthening of the network of existing regulators, whereas the European Parliament (2020b) has proposed the creation of a European Agency for Artificial Intelligence.

I will not comment in detail on this discussion but would like to point to some aspects that should be considered when seeking a way forward. Some structure or body has to take responsibility for many aspects of ethics in AI ecosystems. There must be a place where conceptual positions are collected and defined. The knowledge base and ways of measuring and assessing technologies and their impact need an institutional home, which such a network of existing regulators or a new regulator could offer.

In the UK the non-profit organisation Doteveryone has published a report (Miller and Ohrvik-Stott 2018) on regulating responsible technology which contains a strong analysis of the challenges and proposes the creation of a central hub to guide and support a number of activities. This report employs the ecosystem metaphor of digital technologies and builds on it to explore ways in which entire ecosystems can be governed to serve society. At the core of this report’s recommendations is the creation of what the authors call the Office of Responsible Technology.

The proposed office is explicitly not a regulator for AI. Such a regulator would fall victim to the problem of the lack of clarity in defining AI and might end up as a regulator for everything. Instead, it would be set up as an organisation to support and strengthen existing regulators, such as data protection authorities and financial or other sectoral regulators. These existing regulators are mostly well established and best placed to deal with particular applications, but they often lack knowledge and experience specific to AI or other emerging technologies. The Office for Responsible Technology is therefore described as an organisation that works with regulators and provides the technology-specific knowledge and expertise that they lack.

The Doteveryone report envisages another set of tasks for this office that aligns directly with some of the suggestions I made earlier. It is designated as the home of public engagement, both for the exchange of information and as the place where a vision for technology and society is developed. The report also sees the Office for Responsible Technology as the body responsible for ensuring that redress procedures exist and are usable.

Some aspects of these proposals are debatable. I do not think that all the various tasks proposed for the Office for Responsible Technology need to be located in one organisation. Such a concentration of tasks might make it a large, cumbersome and bureaucratic institution. At the same time it is clear that this idea has traction, as can be seen from the current European discussion of a potential regulator as well as from the fact that organisations are starting to emerge that cover at least parts of this remit, such as the UK’s Centre for Data Ethics and Innovation.^1^

Such an organisation is certainly needed at the political level, whether it be called a regulator, an office, a centre or something else. It should not, however, set out to regulate all of AI, if for no other reason than that it is difficult to define the term. Rather, it should have a remit that covers emerging (digital) technologies and should support existing regulatory structures and processes. This would be a subject of meta-responsibility, i.e. the office would be the organisation responsible for ensuring that technology-related responsibilities are clearly defined and can be fulfilled.

It is worth pointing out that this principle of having a subject of meta-responsibility is not confined to the political level and to national or regional AI ecosystems. A similar organisation or role will be required in other ecosystems, to ensure that there is a mechanism for all ecosystem members to access knowledge, develop capacities, receive guidance and provide input into governance structures. At the level of an organisation this could be a trusted position with responsibility for AI in that organisation. The incumbent could be called the AI officer or, perhaps better, the digital officer. This could be developed in a similar way to the data protection officer, a role that is mandated for European organisations to ensure that data protection requirements are met. The data protection officer works for and is paid by the organisation but has a responsibility defined with regard to data protection requirements, not organisational needs. In the case of a conflict between these, the perspective of the data protection officer is broader than that of the organisation. A similar role with regard to AI could be of crucial importance for the governance of organisational AI ecosystems, which could be a cornerstone of larger and overarching ecosystems. Where appropriate, such roles could also be combined, so that in a company that makes significant use of AI but is not centrally geared towards AI, the data protection officer could simultaneously serve as digital officer.

The suggestions put forward in this section offer examples of the types of actions and interventions that I believe can help move AI ecosystems in a direction conducive to human flourishing, although implementing them will require more thought and detail. The exact form such actions and interventions eventually take will be the subject of further discussion, but my suggestions go some way towards addressing the challenges of AI ecosystems and are consistent with the requirements for interventions that I set out earlier. They could determine the shape of a future-oriented governance framework. Such a framework needs flexibility to ensure that future technologies are accommodated and must open up productive discussion between stakeholders in organisations and countries and internationally to determine how the various AI and related ecosystems are to be developed.

## Footnotes

1 https://www.gov.uk/government/organisations/centre-for-data-ethics-and-innovation
